# Ambulatory Blood Pressure Monitoring: Our Experience in Routine Clinical Practice

**DOI:** 10.7759/cureus.17390

**Published:** 2021-08-23

**Authors:** Sunil R Dube, Manjree Dube, Sayali Damle, Anant Patil

**Affiliations:** 1 Medicine, DY Patil deemed to be University School of Medicine, Navi Mumbai, IND; 2 Family Physician, Shyamlata Clinic, Chembur, Mumbai, IND; 3 Geriatrics, DY Patil deemed to be University School of Medicine, Navi Mumbai, IND; 4 Pharmacology, DY Patil deemed to be University School of Medicine, Navi Mumbai, IND

**Keywords:** ambulatory blood pressure, medical monitoring, masked hypertension, management of hypertension, diabetes type 2

## Abstract

Objective: To evaluate feasibility and usefulness of ambulatory blood pressure monitoring (ABPM) in outpatient setting.

Material and methods: In this prospective study, data of 58 patients who were evaluated with ABPM for diagnosis or therapeutic efficacy purpose were collected from their records. Demographic details of these were recorded. Patients were categorized into different categories based on 24 hours BP pattern. Dipping pattern was compared based on the gender, age, and presence of diabetes or hypertension. Number of patients diagnosed as hypertensive with ABPM reports was compared with office and home BP measurement.

Results: Fifty-eight patients (mean age 57.8 years; 70.69% males) were included of whom 22 (37.93%) underwent ABPM for diagnostic purposes. There was gender-wise significant difference in terms of purpose of performing ABPM (p=0.040). Diabetes was present in 22 (37.93%) patients. Out of 36 known hypertensive patients, 17 (47.22%) patients were receiving dual therapy. Out of 45 patients whose records for active BP variability were available, 26 (57.78%) had high variability. The number and percentage of dippers, extreme dippers and reverse dippers as 23 (42.79%), three (5.56%), and six (11.11%), respectively. Depending on the age, there was significant difference in the dipping pattern (p=0.013). On office blood pressure measurement, 35 (64.81%) patients were found to have hypertension. ABPM revealed hypertension in 32 (59.26%). Masked hypertension and white-coat hypertension was observed in nine (16.17%) and 12 (22.22%) patients, respectively.

Conclusion: ABPM is feasible and useful in routine outpatient clinical practice for diagnosis of essential hypertension, pattern of dipping, masked hypertension, and white-coat hypertension and also for the therapeutic evaluation of patients in clinical practice.

## Introduction

Hypertension is one of the most important chronic healthcare conditions with significant healthcare burden due to its high prevalence and associated complications [1,2]. It is one of the important causes of premature mortality [2]. Regular blood pressure (BP) monitoring helps in early diagnosis and better management of patients with hypertension. Despite several treatment options available, adequate control is still low in many people with hypertension [1]. Traditionally, BP is measured in clinic, often known as office-based BP measurement. Although treatment is often started based on BP measurement performed in the clinic, it does not provide holistic picture of the patient’s BP for 24 hours. The BP in the office may be falsely increased or decreased [2]. Other limitations of office-based measurement include misdiagnosis because of white-coat hypertension or masked hypertension and large variations in small number of BP recordings [3]. Considering these limitations, misclassification of BP status based on the reading taken in the office settings is not uncommon [1].

Ambulatory blood pressure measurement (ABPM) and home BP monitoring, which measures out of clinic BP, are alternative methods for measurement of BPs [1, 4-7]. ABPM can be of diagnostic as well as therapeutic value in the clinical practice [1]. The Spanish Society of Hypertension ABPM registry support its usefulness in the primary care setting for the diagnosis and treatment of hypertension [1]. A multi-center, Indian ABPM study has also demonstrated the usefulness of ABPM for the management of hypertension in Indian patients [8]. A systematic review has suggested that home-based BP monitoring helps in improvement of patient-centered care, control of BP and patient outcomes [9].

Use of 24-hour ABPM is not a new concept. It was introduced about five decades earlier. Initially, the devices were cumbersome to use [10]. Over a period of time, there have been significant developments in the devices and now smaller devices are available for use [10,11]. ABPM has been significantly useful in better understanding of BP behaviors and evaluation of antihypertensive treatment [12].

Despite this, ABPM is overall underutilized in low- and middle-income countries. If used, it can be a very useful complimentary method to office-based evaluation to aid in better diagnosis and therefore better management [4].

ABPM can help in better control of BP, as compared to office monitoring [1]. In our settings, use of ABPM is still not very common. The objective of this study was to evaluate feasibility and usefulness of ABMP in routine clinical practice.

## Materials and methods

In this prospective study, adult patients between 18 and 80 years with BP of 140/90 or more (first time visit or under treatment) were included in the study. Pregnant women, lactating mothers, hospitalized patients, those with atrial fibrillation or having pacemaker implantation, patients with major orthopedic problem in upper limb joint, and those not willing for the procedure were not considered for the study. The enrolled patients were carefully explained the procedure for ABPM. Patients were instructed to sleep on the left side as the device was applied to the right arm. Same investigator explained the procedure to the study participants to avoid any inter-investigator bias. After 24 hours, the readings of ABPM were collected.

Demographic details of the patients, comorbidities, and current antihypertensive drugs (if any) were noted. Prevalence of masked hypertension, white-coat hypertension, and dipping pattern was recorded. Based on diurnal index, the patients were classified as dipping (night-day BP ratio 0.8-0.9), non-dippers (ratio 0.9-1), extreme dippers (ratio <0.8), and reverse dippers (ration >1) [13]. Prevalence of early morning rise in BP and BP variability were noted. The study was approved by independent ethics committee.

Categorical data are presented as frequency and percentages whereas continuous data are presented as mean and standard deviation. Independent samples T test was used for comparison of continuous data between two groups. Non-parametric test (Chi-square test) was used to compare difference in the categorical data between two groups. ANOVA test was used to compare difference in continuous data between multiple groups.

## Results

In our study, a total of 58 patients with mean (SD) age of 57.76 (14.99) were involved. The age range was 26-85 years (Table 1).

**Table 1:** Baseline characteristics (n=58) ABMP: ambulatory blood pressure monitoring

Parameter	Result
Mean (SD) age in years	51.76 (14.99)
Age range in years	26-85
Gender n (%)	
Male	41 (70.69%)
Female	17 (29.31%)
Reasons for ABMP	
Diagnosis	22 (37.93%)
Therapeutic evaluation	36 (62.07%)
Comorbidities	
Yes	33 (56.90%)
No	25 (43.10%)

The mean (SD) age of male and female patients was 50.05 (15.72) and 55.88 (12.54) years, respectively. There was a significant difference in the age of male and female patients (p=0.180).

The study included 41 (70.69%) males and 17 (29.31%) females. In a total of 22 (37.93%) patients ABPM was performed for diagnostic purposes, whereas in 36 (62.07%) patients it was performed for therapeutic evaluation purpose. In 19 (46.34%) male patients ABPM was done for diagnosis, and 22 (53.66%) male patients it was done for evaluation purpose. In three (17.65%) females, ABPM was performed for diagnosis, whereas in 14 (82.35%) female patients, it was performed for evaluation purpose. There was gender-wise significant difference in terms of the purpose of performing ABPM (p=0.040).

A total of 33 (56.90%) patients had some comorbidity. A total of 22 (37.93%) patients had diabetes. Out of them, three patients also had dyslipidemia; two had ischemic heart disease, whereas deep vein thrombosis, obesity, and history of hypoglycemia were also present in one patient each. Other comorbidities included anxiety two (3.45%), hypoglycemia two (3.45%), dyslipidemia three (5.17%), obesity four (6.89%), gastroesophageal reflux disease one (1.72%), and gout one (1.72%).

**Figure 1:**
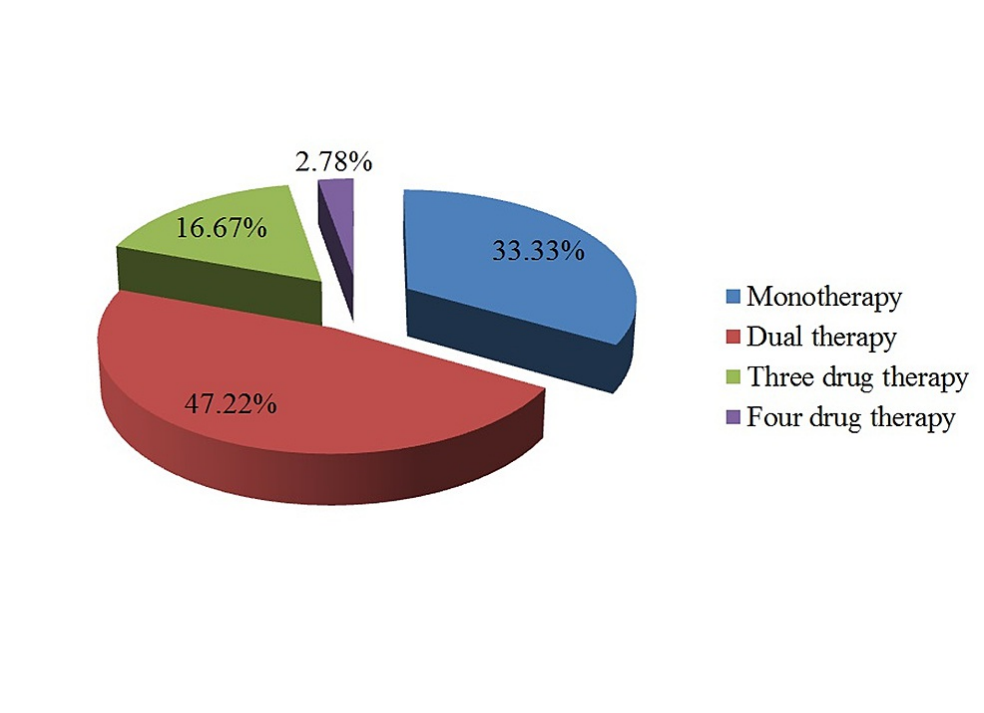
Current medications for treatment for hypertension

Diabetes was present in 12 (29.27%) males and 10 (58.82%) female patients. The difference was statistically significant (p=0.035). Out of 36 known hypertensive patients, 12 (33.33%) were receiving monotherapy for its treatment. Dual therapy, triple therapy, and four-drug therapy was received by 17 (47.22%), six (16.67%), and one (1.72%) patients, respectively (Figure 1).

A total of 38 (65.52%) patients were also receiving other medications. 

**Figure 2:**
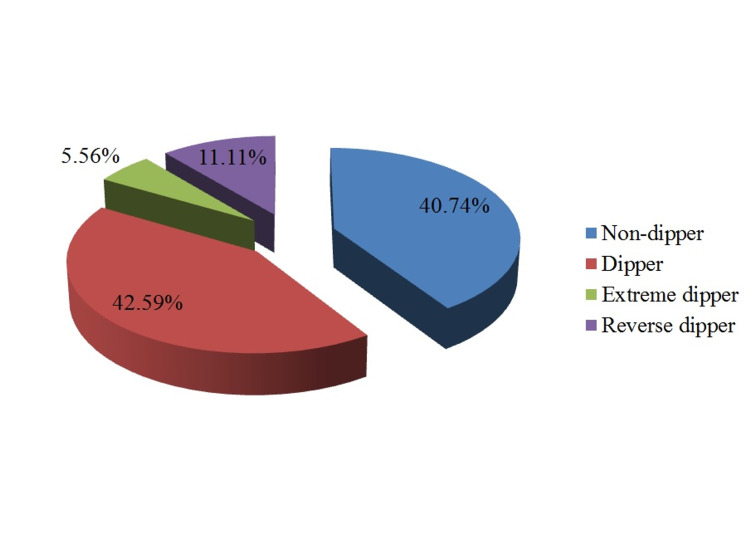
Distribution of patients according to dipping pattern (n=54)

Out of 45 patients whose records for active BP variability were available, 26 (57.78%) had high variability, whereas 19 (42.22%) had normal variability. Out of these 45 patients, passive period systolic blood variability was high 7 (12.1%), whereas diastolic BP variability was high in 37 (63.79%) patients. A total of 22 (40.74%) patients were categorized as non-dippers. The number and percentage of dippers, extreme dippers, and reverse dippers are as 23 (42.79%), three (5.56%), and six (11.11%), respectively (Figure 2).

**Figure 3:**
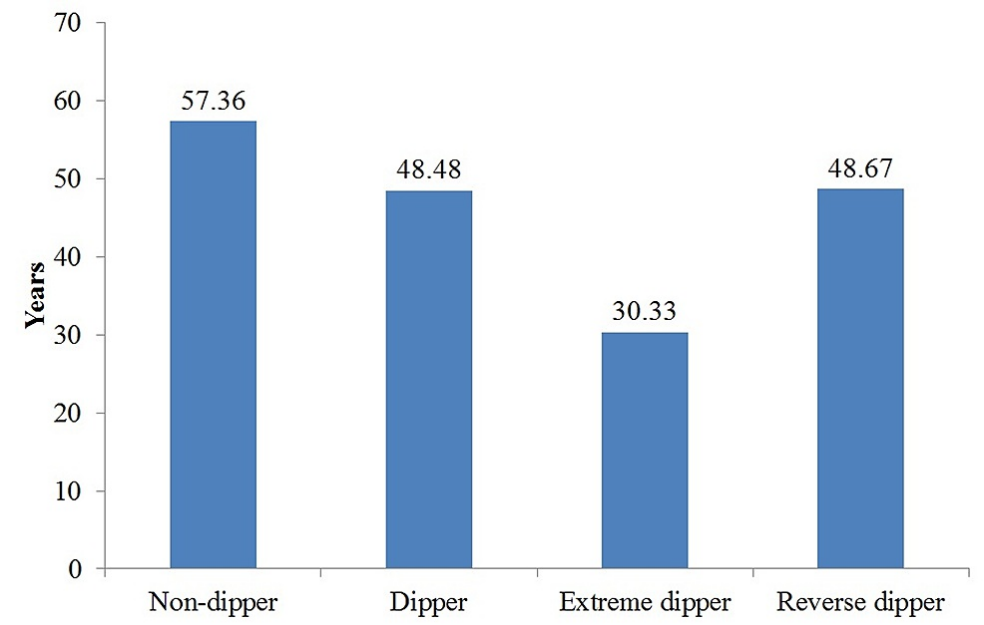
Age of patients according to dipping pattern

The mean (SD) age of non-dippers, dippers, extreme dippers, and reverse dippers was 57.36 (15.94), 48.48 (13.45), 30.33 (4.04), and 48.67 (11.11) years, respectively (Figure 3). 

Depending on the age, there was significant difference in the dipping pattern (ANOVA test p=0.013). Post hoc test showed significant difference in the age of non-dippers versus extreme dippers (p=0.016). In other groups, there was no significant difference.

There was no significant difference in dipping pattern between male and female patients (p=0.108). However, based on the history of hypertension, there was significant difference in dipping pattern (p=0.004). More number of patients without history of hypertension were categorized as extreme dippers and reverse dippers than those with a known history of hypertension. Higher percentage of patients with known hypertension was classified as non-dippers as compared to those without history of hypertension (Table 2).

**Table 2:** Distribution of dipping pattern based on gender, history of diabetes, and hypertension

	Non-dipper	Dipper	Extreme dipper	Reverse dipper	P value
Male (n=37)	14 (37.84%)	18 (48.65%)	3 (8.11%)	2 (5.41%)	0.108
Female (n=17)	8 (47.06%)	5 (29.41%)	0	4 (23.53%)	
Known hypertensive (n=33)	19 (57.58%)	12 (36.36%)	0	2 (6.06%)	0.004
No known hypertension (n=21)	3 (14.29%)	11 (52.38%)	3 (14.29%)	4 (19.05%)	
Diabetes (n=21)	10 (47.62%)	9 (42.86%)	0	2 (9.52%)	0.496
No diabetes (n=33)	12 (36.36%)	14 (42.42%)	3 (9.09%)	4 9 (12.12%)	

Based on the presence of diabetes, there was no difference in dipping pattern (p=0.496).

Out of 50 patients whose data of morning BP were available, 48 (95%) had normal BP, whereas two (4%) patients had high BP. On office BP measurement, 35 (64.81%) patients were found to have hypertension, whereas 19 (35.19%) were normotensive. ABPM revealed hypertension in 32 (59.26%), whereas 22 (40.74%) patients were normotensive (Table 3)

Masked hypertension and white-coat hypertension were observed in nine (16.17%) and 12 (22.22%) patients, respectively. A total of five (13.51%) males had masked hypertension compared to four (23.53%) females. The difference was not statistically significant (p=0.36). White-coat hypertension was present in eight (21.62%) and four (23.53%) male and female patients, respectively. This difference was not statistically significant (p=0.876). Tachycardia was present in a total of 12 (20.69%) patients. Tachycardia was observed in eight (19.51%) and four (23.53%) male and female patients, respectively (p=0.731).

## Discussion

In this study, we evaluated usefulness of ABPM in routine clinical practice at our center. ABPM is required in properly identified patients for correct diagnosis, or modification of antihypertensive treatment. In carefully selected patients, we observed the usefulness of ABPM for diagnosis and evaluation of response to therapy.

One of the indications for performing ABPM is diagnosis of masked hypertension or sleep-time hypertension [14,15]. In fact, it is considered as gold standard for the diagnosis of these conditions [16].

In our study, masked hypertension was observed in 16.17% patients. Office BP measurement may be due to white-coat hypertension [15]. In our study, prevalence of white-coat hypertension was more than masked hypertension. India ABPM study reported white-coat hypertension and masked hypertension prevalence of 12% and 19.3%, respectively. The study reported contradiction of hypertension diagnosis in about one-third of patients with office-based BP measurement versus ABPM [8].

ABPM can also be useful for identification of early morning hypertension and high variability in BP. Both of these hold importance in terms of correlation with target-organ damage and cardiovascular adverse events [15]. In our study, out of 58, readings of morning BP were available for 50 patients. Only two patients among them had high BP in the morning.

Diabetes mellitus and obesity are the known risk factors for cardiovascular complications. These factors are considered as high-risk factors in the management of hypertensive patients. Although well-known for hypertension management, ABPM is still not commonly practiced for the management of hypertension in patients with diabetes and/or obesity. ABPM in these patients may be of use for better risk stratification. Early identification of nocturnal rise is useful for prevention of cardiovascular complications [17]. ABPM can be used for better stratification of patients with diabetes who are susceptible to developing chronic complications [18].

In our study, 37.93% of patients had diabetes. We observed the usefulness of ABPM in patients with diabetes as well as obesity. Non-dipping pattern is common in obese hypertensive people [17]. In our study, overall, 40.74% of patients were classified as non-dippers. Due to a very small percentage of patients with obesity, we did not sub-classify the dipping pattern in obese people. We categorized patients into four categories, i.e., non-dippers, dippers, extreme dippers, and reverse dippers. In our study, there was no difference in dipping pattern in diabetic patients versus non-diabetic patients.

We also evaluated dipping pattern, i.e., nocturnal fall in BP compared to daytime readings and compared it between males versus females, and patients already diagnosed with hypertension versus those who were not diagnosed with hypertension [3]. There was a significant difference in dipping profile of patients based on the diagnosis of hypertension. Higher percentage of known hypertensive patients were classified as non-dippers where more people not diagnosed as hypertensive were categorized as reverse dippers.

There are possibilities that some patients may feel inconvenience during sleep due to intermittent inflation of the cuff at night. We agree to the suggestions provided by others for clinicians to take initiatives to establish ABPM services in their clinical practice, or at least they should access to ABPM elsewhere [14].

Although it is an important measure for diagnosis and management of hypertension, some points should be considered before its application. Large day-to-day variations in the average 24-hour BP may be seen because of genuine biological variability and absence of standardization of activities. Measurement errors can also be possible. Clinicians should know how to perform the procedure and carefully explain it to the patients [19].

A randomized trial has showed that ABPM can also be feasible in pregnancy [20]. It may even be possible in children [21]. However, in our study, pregnant patients or children were not included. Non-comparative study design, single-center data, and limited number of patients are the other limitations of our study. Considering these limitations, we suggest that readers should evaluate and interpret our results appropriately for application in their individual settings.

## Conclusions

ABPM is feasible and useful in routine clinical practice for the diagnosis of essential hypertension, dippers, masked hypertension, and white-coat hypertension. It can also be useful for therapeutic modifications based on the clinical features in the patient.
